# Description of recovery method used for curdlan produced by *Agrobacterium* sp. IFO 13140 and its relation to the morphology and physicochemical and technological properties of the polysaccharide

**DOI:** 10.1371/journal.pone.0171469

**Published:** 2017-02-28

**Authors:** Camila Sampaio Mangolim, Thamara Thaiane da Silva, Vanderson Carvalho Fenelon, Luciana Numata Koga, Sabrina Barbosa de Souza Ferreira, Marcos Luciano Bruschi, Graciette Matioli

**Affiliations:** 1 Postgraduate Program in Food Science, State University of Maringá (UEM), Maringá, Paraná, Brazil; 2 Department of Food Engineering, State University of Maringá (UEM), Maringá, Paraná, Brazil; 3 Postgraduate Program in Pharmaceutical Science, State University of Maringá (UEM), Maringá, Paraná, Brazil; University of Calcutta, INDIA

## Abstract

Curdlan is a linear polysaccharide considered a dietary fiber and with gelation properties. This study evaluated the structure, morphology and the physicochemical and technological properties of curdlan produced by *Agrobacterium* sp. IFO 13140 recovered by pre-gelation and precipitation methods. Commercial curdlan submitted or otherwise to the pre-gelation process was also evaluated. The data obtained from structural analysis revealed a similarity between the curdlan produced by *Agrobacterium* sp. IFO 13140 (recovered by both methods) and the commercial curdlans. The results showed that the curdlans evaluated differed significantly in terms of dispersibility and gelation, and only the pre-gelled ones had significant potential for food application, because this method influence on the size of the particles and in the presence of NaCl. In terms of technological properties, the curdlan produced by *Agrobacterium* sp. IFO 13140 (pre-gelation method) had a greater water and oil holding capacity (64% and 98% greater, respectively) and a greater thickening capacity than the pre-gelled commercial curdlan. The pre-gelled commercial curdlan displayed a greater gelling capacity at 95°C than the others. When applied to food, only the pre-gelled curdlans improved the texture parameters of yogurts and reduced syneresis. The curdlan gels, which are rigid and stable in structure, demonstrated potential for improving the texture of food products, with potential industrial use.

## Introduction

Polysaccharides are an extremely diverse family of natural biopolymers, which are industrially used as thickeners, stabilizers and gelling agents in foodstuffs [1]. There is currently growing interest in their biological functions, such as their antioxidant and prebiotic activity. Although polysaccharides are derived from various sources such as microorganisms, algae and higher plants, the market is dominated by polysaccharides obtained from algae (such as carrageenans, alginates and agar) and higher plants (such as starch, cellulose and pectin) [2].

The synthesis of microbial polysaccharides has emerged as an important source of new biopolymers for industrial use [3]. It is an attractive alternative as microorganisms can grow under controlled conditions and produce a large variety of polysaccharides with unique properties. Production can be carried out in large quantities by biotechnological routes and from renewable and low-cost raw materials, which are easily recovered [4]. One of the microbial polysaccharides of industrial interest is curdlan, a neutral exopolysaccharide composed exclusively of glucose residues joined by β-(1→3) bonds, commercially produced by bacterial species of *Agrobacterium* [2,5–7].

After being discovered by Harada in 1966, curdlan has received considerable attention in both the food and non-food industry due to its physicochemical properties, which are unique when compared to other polysaccharides commonly used, such as starch. When in aqueous suspension, curdlan may form a thermoreversible gel (low-set gel) when heated to temperatures close to 55°C with subsequent cooling. This gel has similar behavior to agar and gelatin gels. In addition, curdlan can form a thermoirreversible gel (high-set gel) when its aqueous suspension is heated to temperatures above 80°C, which is very stable. Furthermore, gels with different strengths can be formed by varying the temperature, the heating time and the concentration of curdlan [1,6].

Curdlan was approved for use in foodstuffs in Korea, Taiwan and Japan in 1989 and registered in 1996 by the FDA (*Food and Drug Administration*) in the USA as a food additive with the following functions: a formulation and processing aid, and a stabilizer, thickener and texturizer [8]. It is widely used due to its ability to form the thermoirreversible gel, which exhibits great stability during the industrial processes of autoclaving, frying and freezing-thawing cycles. It has no taste, color or odor, can form gels at a wide pH range (from 2 to 10) and can mimic the palatability of foods containing fat [9]. It is also considered a dietary fiber, thus improving the functionality of various food products. The versatility of its applications associated with its health benefits make curdlan a valuable tool in the development of innovative food systems [1,3].

The dispersion and gelation characteristics of curdlan in an aqueous system, as well as its mechanical properties, are associated with several factors such as concentration, temperature, heating time, dispersal method, and the presence of ions, salts and low molecular weight sugars [10]. Therefore, the form of recovery from the culture medium and the purification of the polysaccharide are steps which decisively influence the physical and technological properties of curdlan, which directly affect the sensory characteristics of foods in which the polysaccharide is inserted. So, it is necessary to study these properties to define the most appropriate form of the implementation of the polysaccharide in the industry [11].

Considering the interest in the properties of dispersion, viscosity, gelation and the health benefits of curdlan, the present study aimed to characterize the physicochemical and technological properties of curdlan produced by *Agrobacterium* sp. IFO 13140 and recovered using the pre-gelation and precipitation methods, as well as commercial curdlan subjected or otherwise to pre-gelation treatment. The methodologies employed for the recovery of the curdlans were studied. The structures of all curdlans were compared by FT-Raman spectroscopy. In addition, the application of curdlans in yogurts was evaluated.

## Materials and methods

### Materials

The bacterial strain *Agrobacterium* sp. IFO 13140 was purchased in lyophilized form from the Institute for Fermentation of Osaka (Japan). Commercial curdlan was acquired from Wako Pure Chemical Industries, Ltd. (Osaka, Japan). All solvents were of analytical grade.

### Curdlan production by *Agrobacterium* sp. IFO 13140

The culture medium used to reactivate the microorganism was proposed by the supplier (g L^-1^), pH 7: polypeptone (10), yeast extract (2), MgSO_4_.7H_2_O (1). 30 mg of the lyophilized bacteria were incubated in 100 mL of the medium at 30°C and 120 rpm for 48 h. The cells were recovered through centrifugation (6000 ×g, 10 min), washed with 9 g L^-1^ NaCl and transferred to the production medium. For curdlan production, the liquid medium described by Martinez et al. [12], pH 7, was used (g L^-1^): glucose (50), KH_2_PO_4_ (2.7), NH_4_Cl (1.6), MgSO_4_ (0.5) and trace elements (10 mL L^-1^). The composition of the trace elements (g L^-1^) in HCl 0.1 mol L^-1^ was: FeCl_3_.6H_2_O (1), MnCl_2_.4H_2_O (1), ZnCl (1), CaCl_2_ (1) and CaCO_3_ (0.03). The reactivated microorganisms were transferred to Erlenmeyer flasks containing 100 mL of the production medium and maintained at 30°C and 150 rpm for 5 days.

### Recovery of curdlan from production medium

Two methodologies were used to recover curdlan from the medium. In pre-gelation method [12,13], NaOH 3 mol L^-1^ was added to the Erlenmeyer flasks containing the production medium at a ratio of 1.8:1 (NaOH:medium) for curdlan solubilization. This mixture was centrifuged (18000 ×g, 15 min, 4°C) to separate the cells. HCl 3 mol L^-1^ was added to the supernatant until pH 6–7 to obtain the curdlan gel, which was recovered by centrifugation (18000 ×g, 15 min, 4°C). Subsequently, it was washed three times with distilled water and lyophilized. The commercial curdlan was subjected to the same treatment and it was named pre-gelled commercial curdlan.

The precipitation method is an adaptation of the industrial purification of curdlan. Industrially produced curdlan is purified by dissolution in a strong alkaline solution and dried in a spray-dryer, then washed with water until neutralization [14]. NaOH 3 mol L^-1^ was added to the Erlenmeyer flasks containing the production medium at a ratio of 1.8:1 (NaOH: medium) to solubilize the curdlan and the mixture was centrifuged at 18000 ×g for 15 min at 4°C to separate the cells, before being lyophilized. The dried mixture was resuspended in water, filtered to remove larger impurities and then ultra-filtered using the system described by Fenelon et al. [15] with a 30 kDa membrane and pressure of 1 kgf cm^-2^. During ultrafiltration, the material was washed with water until the pH was neutralized, causing the precipitation of the curdlan, that was thereafter lyophilized.

### Structural analysis of curdlans by FT-Raman

Samples of the commercial and commercial pre-gelled curdlans and those produced by *Agrobacterium* sp. IFO 13140 (pre-gelation and precipitation methods) were analyzed by Fourier transform Raman scattering infrared spectroscopy (FT-Raman) using a Fourier Transform infrared spectrometer (Vertex 70v model with Ram module II, Bruker, Germany) equipped with a Germanium detector cooled with liquid nitrogen. A Nd:YAG laser was used for excitation at 1064 nm. The spectra were based an average of 200 scans with a resolution of 4 cm^-1^.

### Morphology of curdlans

The morphology of the commercial and commercial pre-gelled curdlans, and the curdlans produced by *Agrobacterium* sp. IFO 13140 (pre-gelation and precipitation methods) was analyzed in a scanning electron microscope (SS-550 model, Superscan, Shimadzu, Japan), at an accelerating voltage of 15 kV.

### Physicochemical characterization of curdlans

The commercial and commercial pre-gelled curdlans and those produced by *Agrobacterium* sp. IFO 13140 (pre-gelation and precipitation methods) were characterized for their carbohydrate, moisture and sodium content. The carbohydrate content was determined through the phenol-sulfuric method [16]. The moisture content was determined by the gravimetric method [17] and sodium was identified using atomic absorption spectrometry [18].

### Technological properties of curdlans

#### Water dispersion and gel formation capacities of curdlans

To verify the dispersal capacity in water of the commercial and commercial pre-gelled curdlans and those produced by *Agrobacterium* sp. IFO 13140 (pre-gelation and precipitation methods), they were subjected to stirring in a mixer and/or in a magnetic stirrer until homogeneous dispersion was achieved. The dispersions were heated at 95°C/1 h to assess the gel formation capacity.

#### Rheological characteristics and gel strength of curdlans

Dispersions of the pre-gelled commercial curdlan and the curdlan produced by *Agrobacterium* sp. IFO 13140 (pre-gelation method) were prepared at three concentrations: 20 g L^-1^, 40 g L^-1^ and 80 g L^-1^ in water. The curdlans were dispersed in water using a mixer at room temperature for 5 min, sonicated for 10 min and then stirred in a magnetic stirrer for 12 h at 40°C. The samples were analyzed in a controlled stress rotational rheometer (HAAKE MARS II model, Thermo Fisher Scientific Inc., Newington, Germany), with steel cone/plate geometry (60 mm, gap 0.052 mm). The elastic (G') and viscous (G") modulus, and the apparent viscosity were measured depending on temperature (20–60°C) at a frequency of 10 Hz. For gel strength evaluation, the dispersions of both samples at the concentration of 20 g L^-1^ were kept or not in a water bath at 61°C/1 h to prepare the low-set gel and at 95°C/1 h for preparation of the high-set gel [19]. After heating, they were cooled at room temperature. The strength of the suspensions and gels was evaluated in triplicate in a Stable Micro Systems texturometer (TA-XT Plus model, Texture Technologies Corp., UK), using a 36 mm probe for compression analysis.

#### Water Holding Capacity (WHC), Oil Holding Capacity (OHC) and Water Solubility Index (WSI)

Samples of 0.25 g of the commercial and pre-gelled commercial curdlans and curdlan produced by *Agrobacterium* sp. IFO 13140 (pre-gelation method) were diluted in 10 mL of distilled water or soya oil at 30°C, homogenized in a magnetic stirrer for 30 min and centrifuged (1500 ×g, 10 min). The WHC was expressed as g of water absorbed per g of curdlan sample and the OHC was described as g of oil absorbed per g of curdlan sample. To determine the WSI, the supernatant of the WRI analysis was oven dried at 105°C and the ratio between the weight of the solid residue present in the supernatant after drying and the weight of the curdlan sample was calculated [11].

### Application of curdlan in yogurt

#### Preparation of yogurts

Eight yogurt samples were prepared: A1) without curdlan or heat treatment; A2) without curdlan and with heat treatment; B1) with commercial curdlan and without heat treatment; B2) with commercial curdlan and heat treatment; C1) with pre-gelled commercial curdlan and without heat treatment; C2) with pre-gelled commercial curdlan and heat treatment; D1) with curdlan produced by *Agrobacterium* sp. IFO 13140 (pre-gelation method) without heat treatment; D2) with curdlan produced by *Agrobacterium* sp. IFO 13140 (pre-gelation method) with heat treatment.

The curdlan was mixed in UHT (ultra-high temperature) whole milk at a concentration of 15 g L^-1^ using a mixer. Subsequently, the milk of the samples subjected to heat treatment was heated at 90°C for 120 seconds and cooled to 42°C. For samples without heat treatment, the milk was directly heated to 42°C. Next, all samples were inoculated with 2 g L^-1^ of mixed culture of *Streptococcus thermophilus* and *Lactobacillus delbrueckii* subsp. *bulgaricus*, and maintained at 42°C for 6 h. The yogurts were cooled and refrigerated for 24 h for rheological, texture and syneresis analysis [3,20].

#### Texture Profile Analysis (TPA)

The TPA of the yogurts was carried out in a TA-XT Plus texturometer (Stable Micro Systems, Godalming, UK), equipped with Texture Expert software (Stable Micro Systems, Godalming, UK). A 10 mm cylindrical probe was used (ref. P/0.5R, Stable Micro Systems). Two cycles were applied, at a velocity of 2 mm s^-1^ and a depth of 15 mm, producing force-time curves which were used to determine the firmness, cohesiveness, adhesiveness, springiness, gumminess and chewiness of the samples. The analysis was performed in triplicate, at 8°C.

#### Syneresis

The syneresis analysis of each preparation was performed after 28 days of storage of the yogurt at 8°C in triplicate. Yogurt samples (30–40 g) were centrifuged (222 ×g, 10 min, 4°C) and the supernatant was separated and centrifuged again (222 ×g, 10 min, 4°C), weighed and the syneresis (%) was calculated from the ratio between the mass of the supernatant and the initial mass of the yogurt [20].

#### Rheological analysis

The rheological properties of flow of the yogurts were analyzed in triplicate, in a controlled stress rotational rheometer (HAAKE MARS II model, Thermo Fisher Scientific Inc., Newington, Germany), with steel cone/plate geometry (60 mm, gap 0.052 mm). The measurements were performed at 8°C. The scan of the deformation rate was performed from 0 to 116 s^-1^, obtaining the outward and return data. The flow and viscosity curves were obtained by determining the stress and viscosity versus the shear rate [20]. The rheological parameters K (consistency index) and n (flow behavior index) were calculated using the Ostwald de Waele model, while the parameter τ_0_ (yield stress) was calculated by the Herschel-Bulkley model. The hysteresis was calculated from the area of the flow curves of the yogurts using the RheoWin 4.10.000 software program (HAAKE software, Thermo Fisher Scientific Inc., Newington, Germany).

### Statistical analysis

Data were analyzed by analysis of variance (ANOVA), and means were compared with the Tukey Test (p<0.05) using the Statistica 8.0/2008 software package (Stat Soft, Inc., Tulsa, USA).

## Results and discussion

### Structural analysis of curdlans by FT-Raman

**Fig 1:**
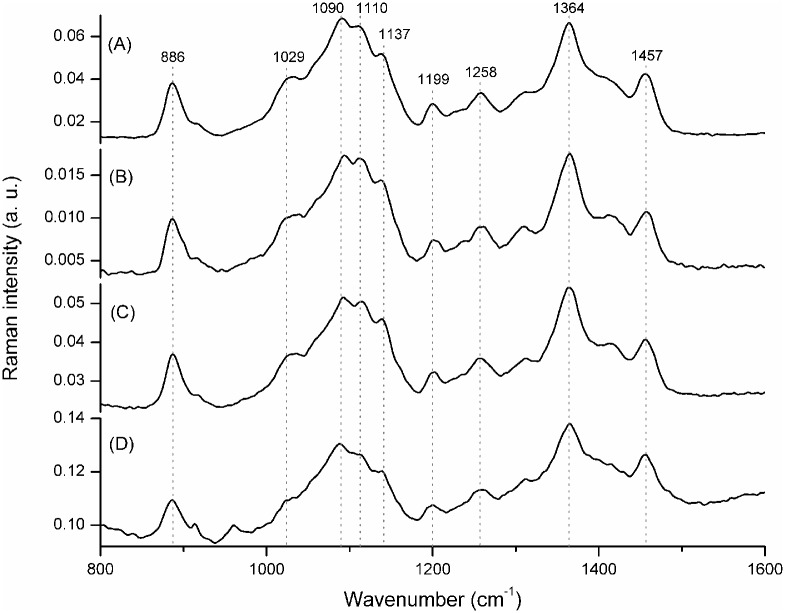
FT-Raman spectra of: (A) commercial curdlan, (B) pre-gelled commercial curdlan, (C) curdlan produced by *Agrobacterium* sp. IFO 13140 (pre-gellation method), (D) curdlan produced by *Agrobacterium* sp. IFO 13140 (precipitation method). The dotted lines show the characteristic peaks of the samples.

Fig 1 shows FT-Raman spectra of commercial and pre-gelled commercial curdlans and those produced by *Agrobacterium* sp. IFO 13140 (pre-gelation and precipitation methods). From the data, it is verified a structural similarity between all samples, which means that the curdlan produced by the microorganism has a similar structure to commercial curdlan and it also means that the method employed to recover curdlan from the medium did not modify the structure of the polysaccharide.

Several authors described the chemical bonds present in the structure of curdlan molecule based in FT-IR analysis [3,14,21]. They stated that, from the FT-IR data for curdlan, the bands around 890, 1080 and 1160 cm^−1^ correspond to the *β*-(1,3)-glucan linkages. Despite the difference in band assignments for FT-IR and FT-Raman spectroscopies, the spectra of carbohydrates in both techniques show a characteristic absorption band of β-anomeric configuration at ~890 cm^−1^ (886 cm^-1^ in this work) [22]. The Raman bands and shoulders at 1090 and 1137 cm^−1^ are typical for β-glucans. Intense highly overlapped Raman bands between 990 and 1200 cm^−1^ are attributed to COC and CC stretching vibrations of polysaccharides. The features between 1200 and 1440 cm^-1^ are mainly assigned to in-plane ring deformation including CH and OH bending modes. Finally, the band at 1457 cm^-1^ is assigned to CH_2_ in-plane bending in CH_2_OH of the molecule [23].

### Morphology of curdlans

**Fig 2:**
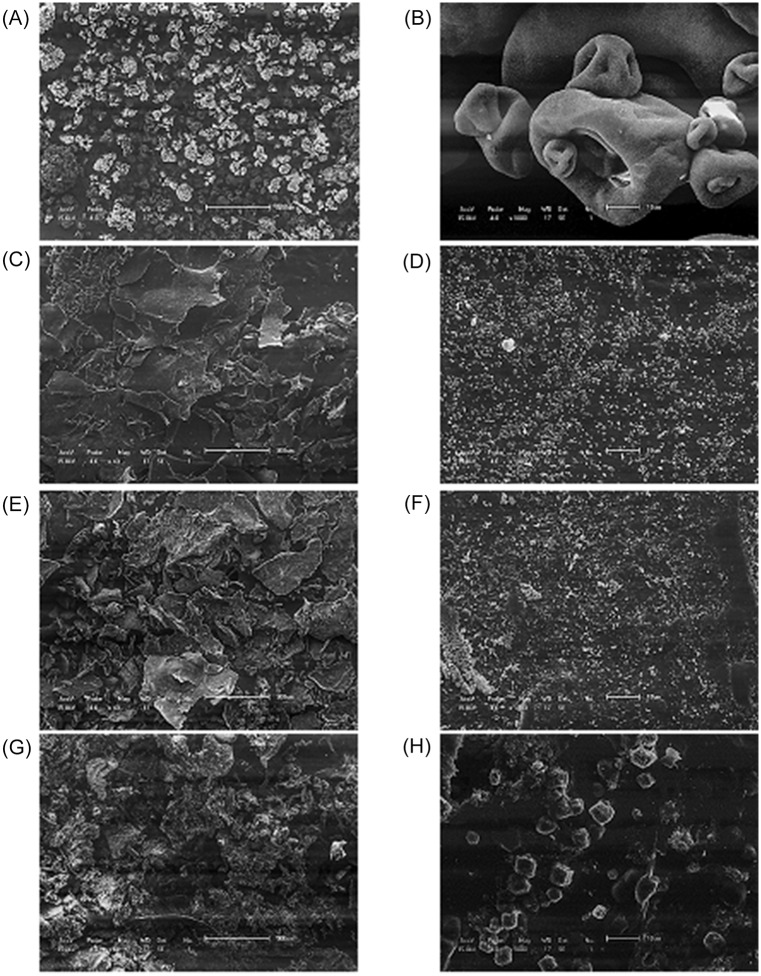
Scanning electron microscopy of: commercial curdlan—(A) 40x and (B) 1000x magnification; pre-gelled commercial curdlan—(C) 40x and (D) 1000x magnification; curdlan produced by *Agrobacterium* sp. IFO 13140 (pre-gelation method)—(E) 40x and (F) 1000x magnification; curdlan produced by *Agrobacterium* sp. IFO 13140 (precipitation method)—(G) 40x and (H) 1000x magnification.

Fig 2 shows scanning electron microscopy images of the commercial curdlan (Fig 2A and 2B), the pre-gelled commercial curdlan (Fig 2C and 2D) and the curdlans produced by *Agrobacterium* sp. IFO 13140 recovered by pre-gelation and precipitation methods (Fig 2E–2H) with a range of magnifications.

Marchessault and Deslandes [24] define the shape of the granules of commercial curdlan as collapsed or invaginated, which coincides with the commercial curdlan shape illustrated in Fig 2A and 2B. The granules have widely differing sizes, varying from around 10 to 100 μm in diameter. Industrially produced curdlan is dried by spray-drying [14] and the type of drying has a major influence on the particle bead structure [25]. The expected range of particle size of polymers dried by ordinary spray dryers is 5–150 μm. Furthermore, spherical microspheres or those with pores/concavities, as shown in Fig 2A and 2B, are characteristic of the drying of products by a spray-dryer. The formation of pores or concavities is associated with the rapid evaporation of the liquid particles of this process [26].

The pre-gelled commercial curdlan and both the curdlans produced by the microorganism, when viewed at low magnification (40x), were in flake form and displayed irregularities. These characteristics are expected for lyophilized products, since the working conditions of the lyophilizer exert great pressure on the particles to be dried, meaning that a product dried by lyophilization remains amorphous in comparison with a spray dried product [25]. Furthermore, at a higher magnification (1000x), it can be seen that the particles obtained for the three samples that underwent the lyophilization process (Fig 2D, 2F and 2H), were considerably smaller than the commercial curdlan (obtained by spray-dryer). Therefore, it can be inferred that the lyophilization process can facilitate the dispersion of curdlan molecules in water, especially the pre-gelled commercial curdlan and the one produced by *Agrobacterium* sp. IFO 13140 (pre-gelation method), which have smaller particle sizes (Fig 2D and 2F). The smaller diameter increases the accessibility of water molecules to the inside of the particles, facilitating dispersion.

The structure displayed by the commercial curdlan is maintained by a large amount of hydrogen bonds. But, when added to an alkaline solution, these bonds are broken due to their ionization, and the granule loses its structure. The neutralization of the curdlan suspension causes a reshaping of the hydrogen bonds that depends on the neutralizing agent used. With the addition of HCl (pre-gelation method), the reassociation causes the formation of a firm gel, with small structures of less than 1 μm in diameter (Fig 2D and 2F). With water (precipitation method), reassociation causes the precipitation of the curdlan into small particles with diameters greater than those obtained by neutralization with HCl, ranging from 2–10 μm in diameter (Fig 2H). Therefore, the difference between the sizes of the curdlan particles produced by the microorganism using the two methods is due to the different forms of reassociation of the hydrogen bonds, which are dependent on the recovery method employed, and can also influences the characteristics of water dispersion of the polysaccharide.

### Physicochemical characterization of curdlans

As an exopolysaccharide, curdlan is secreted to the extracellular medium in the form of biofilms, and has the advantage of being easy to recover. As a result, the polysaccharide should present a low degree of impurities, and consequently the carbohydrate content represents an indirect measure of the purity of the samples. The levels of carbohydrates and moisture found in the curdlan samples are shown in Table 1.

**Table 1:** Carbohydrate, moisture and sodium content (%) of different samples of curdlan. Values indicate mean ± standard-deviation.

Curdlan sample	Carbohydrate	Moisture	Sodium
Commercial	94.5 ± 0.2	4.1 ± 0.4	1.2 ± 0.3
Commercial pre-gelled	82 ± 1	4.0 ± 0.3	4.4 ± 0.8
Microbial* (pre-gelation method)	70.6 ± 0.6	5.4 ± 0.9	0.024 ± 0.001
Microbial* (precipitation method)	85.4 ± 0.7	4.8 ± 0.6	< 0.001

*Produced by *Agrobacterium* sp. IFO 13140.

The highest carbohydrate content was found for the commercial sample. The pre-gelled commercial sample had a carbohydrate content lower than the commercial curdlan due to the salt (NaCl) incorporated in the pre-gelling process. The two samples produced by *Agrobacterium* sp. IFO 13140 had different carbohydrate contents, with that produced by precipitation method having greater purity due to the successive washings performed. The impurities in the last samples consist of remnants of the production medium and the salt formed in the recovery step.

The sodium content of curdlans was determined because curdlan is a glucose polymer with a low amount of inorganic salts, mainly sodium chloride [27]. Thus, sodium content is an indirect estimate of the salt content of the material. The sodium concentrations disposed in Table 1 directly influence the dispersion and gelling characteristics of the polysaccharide, which will be further discussed in the next section.

### Technological properties

#### Water dispersibility and gelling capacities

Curdlan can be used as a thickener, stabilizer and texturizer in food industry [8,27]. However, as curdlan is insoluble in water, it is necessary to use an efficient homogenizer to obtain a homogeneous dispersion and apply a further stirring of the dispersion prior to the carrying out of analyses requiring uniformity [21].

The pre-gelled commercial curdlan and the curdlan produced by *Agrobacterium* sp. IFO 13140 (pre-gelation method) dispersed easily in water when subjected to stirring in a mixer for less than 5 min, and also acted as thickening agents. Both dispersions remained visibly homogeneous for days and, when heated at 95°C/1 h, formed a firm and homogeneous gel. The commercial curdlan did not easily disperse in water using the mixer. After 24 hours of stirring in a magnetic stirrer, a homogeneous dispersion formed for a brief period, displaying a phase separation after 10 min of rest. After 48 h of stirring, phase separation began to be observed at 30 min; after 72 h, at 1 hour; and after 96 h, phase separation began at 1.5 h. The gelling of commercial curdlan also occurred after heating at 95°C/1 h, but there was phase separation in the gel formed, which became more pronounced as the stirring period for formation of the dispersion was reduced. Marchessault and Deslandes [24] have previously identified the formation of a non-homogenous gel from curdlan in its native form. Probably, the difficulty of dispersion of commercial curdlan compared with those submitted to pre-gelation process is related to the particle size of the sample (as noted in morphological analysis), which makes difficult the accessibility of water molecules to the inside of the particles.

Another important factor related to the dispersion and gelling of curdlan is the presence of salts. In the gelling of curdlan, the swelling phase of the molecule is essential and may be influenced by salts, as they affect the mobility of the water molecules during hydration [10]. The swelling of curdlan is facilitated with the salts present because it increases intermolecular association and consequently increases the viscosity of curdlan water dispersions. The curdlan produced by *Agrobacterium* sp. IFO 13140 (precipitation method) displayed greater difficulty in forming a homogenous dispersion in water and, in contrast to the other curdlans, did not form a gel when heated. This can be explained by its considerably lower sodium content, due to the countless washings with water used to achieve neutralization. Samples of the other curdlans were also washed in the ultrafiltration device and, following such process, no longer had the same dispersion and gelling capacities. This proves therefore, that as with other biopolymers, for curdlan, the rheological characteristics depend excessively on the methods of recovery employed following production. Due to the characteristics displayed, the technological characteristics of the curdlan produced by *Agrobacterium* sp. IFO 13140 (precipitation method) were not evaluated, and this sample was not applied in yogurt. Additionally, dispersions of commercial curdlan were not evaluated by rheological and gel strength analysis because the commercial curdlan did not act as a thickener with the methodology employed for preparation of the dispersions.

#### Rheological characteristics and gel strength of curdlans

**Fig 3:**
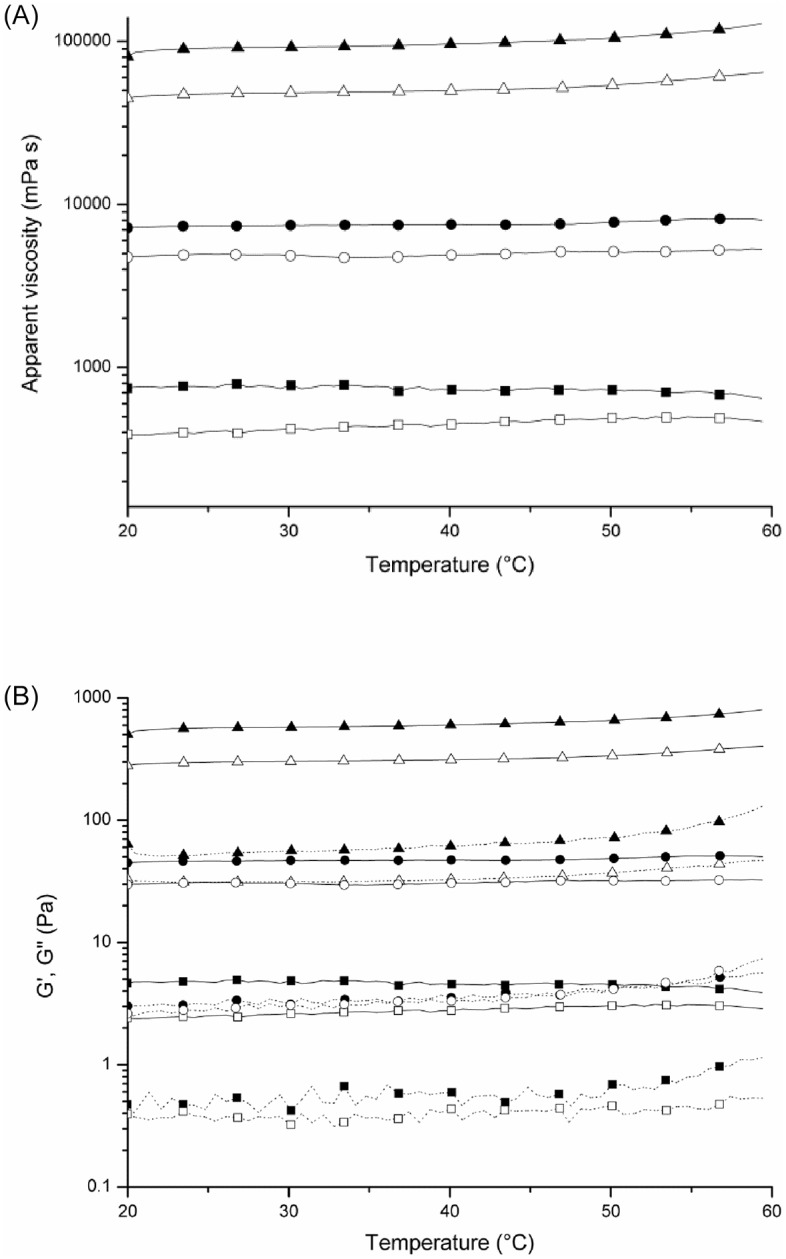
Temperature dependency of: (A) apparent viscosity and (B) G' (continuous line) and G" (dotted line) modulus of the aqueous dispersions of curdlan. Pre-gelled commercial curdlan (empty symbol) and curdlan produced by *Agrobacterium* sp. IFO 13140 recovered by the pre-gelation method (full symbol) at (■) 20, (●) 40 and (▲) 80 g L^-1^.

Thermal scanning rheological measurements were made to evaluate both the apparent viscosity and the gelation behavior of curdlan dispersions. The temperature dependence of apparent viscosity and of G' and G" modulus for aqueous dispersions of pre-gelled commercial curdlan and of the curdlan produced by *Agrobacterium* sp. IFO 13140 (pre-gelation method) at 20, 40 and 80 g L^-1^ are shown in Fig 3A and 3B.

The curdlan produced by *Agrobacterium* sp. IFO 13140 (pre-gelation method) exhibited considerably higher viscosity than pre-gelled commercial curdlan (Fig 3A). For example, at 20°C, the apparent viscosity of curdlan produced by the microorganism was almost 100% higher than pre-gelled commercial curdlan at 20 and 80 g L^-1^. Equally, G' and G" modulus (Fig 3B) were also higher for the curdlan produced by the microorganism, revealing the highest thickening potential of this curdlan when compared to the pre-gelled commercial one.

Both the apparent viscosity and the G' module of both curdlans exhibited a different behavior as a function of temperature for each concentration employed. At 20 g L^-1^, both parameters increased with temperature for pre-gelled comercial curdlan, while for curdlan produced by the microorganism they decreased. This behavior is related to the swelling of curdlan because of the breakage of hydrogen bonds during heating. The difference between the curdlanas is because, for pre-gelled commercial curdlan, inter-molecular entanglements between the particles that were swollen formed pseudo-links, which contributed to the little increase in G'. This phenomenon did not occur for curdlan produced by the microorganism. However, for higher concentrations (40 and 80 g L^-1^), the decrease in the viscosity and in G' no longer occurred with temperature for any sample, because the high concentration favors the inter-molecular entanglements between the particles, which contribute to the increase of both parameters. Additionally, the increase in G' and G" modulus and in apparent viscosity of curdlan dispersions at about 50–55°C, especially at 80 g L^-1^, suggests the beginning of the formation of the thermo-irreversible gels due to hydrophobic interactions.

Jin et al. [21] stated that G' decreased until 50°C for curdlan suspensions at 20 g L^-1^, which is related to the swelling of curdlan because of the breakage of hydrogen bonds, as observed for curdlan produced by *Agrobacterium* sp. IFO 13140 (pre-gelation method) in this study. However, Funami et al. [28] stated only increasing in G' parameter for dispersions of curdlans at 20, 40 and 100 g L^-1^ with increasing temperature. The authors attributed this behavior to inter-molecular entanglements between the particles that were swollen forming pseudo-links, being this result similar to the one obtained for pre-gelled comercial curdlan in all concentrations evaluated and for curdlan produced by the microorganism for concentrations from 40 to 80 g L^-1^ in this work.

The strength data of suspensions and gels of the pre-gelled commercial curdlan and the curdlan produced by *Agrobacterium* sp. IFO 13140 (pre-gelation method) at 20 g L^-1^ are presented in Table 2. According to the strength values of the suspensions and gels, the curdlan produced by *Agrobacterium* sp. IFO 13140 (pre-gelation method) has a better thickening capacity than the pre-gelled curdlan, as the suspension of the same without heat treatment was approximately 10% stronger. This result agrees with the results of rheological analysis. However, the gelling capacity of the pre-gelled commercial curdlan is considerably higher.

**Table 2:** Strength (×10^−3^ N) of pre-gelled commercial curdlan and the curdlan produced by *Agrobacterium* sp. IFO 13140 (pre-gelation method) samples after undergoing different heat treatments. Values indicate mean ± standard-deviation.

Heat treatment of the dispersion in a concentration of 20 g L^-1^	Strength (× 10^−3^ N)	
	Pre-gelled comercial curdlan	Curdlan from microorganism (pre-gelation method)
Without heat treatment	69 ± 2^d^	76 ± 2^c^
Low-set gel (61°C/1 h)	79 ± 1^c^	77 ± 2^c^
High-set gel (95°C/1 h)	96.8 ± 0.8^a^	83 ± 1^b^

^a–d^ Means with different letters are significantly different (p< 0.05).

The strength of the low-set gel of the pre-gelled commercial curdlan was around 15% greater than that of the suspension without heat treatment, and statistically equal to that of the low-set gel of the curdlan produced by the microorganism. The high-set gel of the pre-gelled commercial curdlan had strength 40% greater than the suspension without heat treatment, and 17% greater than the high-set gel produced by the microorganism.

When an aqueous dispersion of curdlan is heated around 55°C, the low-set gel formed is maintained by intramolecular hydrogen bonds and the curdlan chains adopt a predominantly single helix conformation. But when heated above 80°C, the high-set gel formed is maintained by intermolecular hydrophobic interactions and the curdlan chains adopt a predominantly triple helix conformation [14,29], forming an organized and rigid gel configuration. As such, the increase in the strength of the high-set curdlan gel (95°C/1 h) is due to the greater presence of the triple helix conformation.

The fact that the curdlan produced by *Agrobacterium* sp. IFO 13140 (pre-gelation method) have formed a considerably weaker gel than the pre-gelled commercial curdlan, as well as the fact that it does not exhibit the same behavior that pre-gelled commercial sample in rheological analysis at low concentrations (increase of G' and apparent viscosity with temperature), are probably due to a difference of molecular weight/degree of polymerization of the polymers. A variety of physical properties of β-(1→3)-glucans, including gel strength (or gel-forming ability) is related to the molecular weight/degree of polymerization of the biopolymer. The higher the degree of polymerization of the polysaccharide, the greater is its gel forming ability with heating [30,31].

Nakanishi et al. [32] studied the formation of a complex of curdlan with aniline blue dye and noted a relation between the rate of interaction of the polymer with the dye and the concentration, degree of polymerization, and gel-forming ability of the polymer. The variation in absorbance at 590 nm is proportional to the concentration of curdlan and to its gel-forming ability (and consequently to its degree of polymerization). S1 Appendix contains the methodology used by the authors described to evaluate curdlan interaction with aniline blue dye and the results of the relationship between absorbance variation and concentration of the pre-gelled commercial curdlan and curdlan produced by the microorganism. By comparing the results of this study to those obtained by Nakanishi et al. [32] and using the relation obtained by these authors between absorbance variation and degree of polymerization, it is concluded that the degree of polymerization of the pre-gelled commercial curdlan is about 45% higher than of the curdlan produced by the microorganism, which explains the lowest gel strength and the decrease in apparent viscosity and G' in low concentration dispersions of the polymer.

#### Water Holding Capacity (WHC), Oil Holding Capacity (OHC) and Water Solubility Index (WSI)

Determining the technological properties of polysaccharides is of great importance when predicting their possible industrial applications. The values for the properties of water solubility index (WSI) water holding capacity (WHC) and oil holding capacity (OHC) of the curdlan samples are described in Table 3.

**Table 3:** Water Holding Capacity (WHC), Oil Holding Capacity (OHC) and Water Solubility Index (WSI) (g g^-1^) of the commercial, pre-gelled commercial curdlans and those produced by *Agrobacterium* sp. IFO 13140 (pre-gelation method). Values indicate mean ± standard-deviation.

Curdlan sample	WHC	OHC	WSI
Commercial	4.6 ± 0.4^a^	0.62 ± 0.08^c^	0.006 ± 0.002^a^
Commercial pre-gelled	2.20 ± 0.08^c^	4.4 ± 0.2^b^	0.00923 ± 7E-5^a^
Microbial (pre-gelation method)	3.6 ± 0.3^b^	8.7 ± 0.1^a^	0.0068 ± 0.003^a^

^a–c^ Means in the same column with different letters are significantly different (p< 0.05).

The three curdlans had very low and statistically equal WSIs, which was expected as curdlan is insoluble in water. Despite this, however, the curdlans presented some water absorption, with the commercial curdlan achieving the highest value, followed by the curdlan produced by the microorganism and the pre-gelled commercial curdlan. Seguchi and Kusunose [33] found water absorption rates for curdlan between 5.244 g g^-1^ and 7.724 g g^-1^.

Compared to other polysaccharides employed in food industry as gums, curdlan has low water holding capacity; the guar and xanthan gums have a WHC of 25.77 g g^-1^ and 27.33 g g^-1^, respectively [34]. However, both are soluble in water. The low solubility and water holding capacity of curdlans is explained by the existence of a large amount of intra/intermolecular hydrogen bonds within the polymer. This also explains the fact that commercial curdlan has a higher water absorption index than the other types as the recovery by the pre-gelation process employed in the latter two types favors the formation of large quantities of hydrogen bonds in the polymer. Thus, the polysaccharide interacts more strongly with itself than with water [21,34].

The three evaluated curdlans also differed with respect to oil holding capacity. With the exception of the commercial curdlan, they presented greater OHC values than WHC, indicating that the samples have a higher lipolytic than hydrophilic capacity. Oil absorption values greater than three make curdlan a potentially useful ingredient in structural interactions in foods, especially in aroma retention, improved palatability and maintenance of the stability of the product during storage [11]. This result justifies the use of curdlan as a fat replacer or mimicker of fat in the food industry [9,35].

### Application of curdlan in yogurt

The main characteristics that define the quality of yogurts are its texture and propensity for serum separation (syneresis). Typically, polysaccharides such as xanthan, guar, gellan, pectin, carrageenan are used to give the product a firmer texture, increase its stability and hence make it more acceptable to the consumer [3,20]. Table 4 displays the parameters of texture and syneresis after 28 days of storage of yogurt samples with and without curdlan, submitted or not to heat treatment with the aim of gelling the curdlan in the milk prior to the fermentation process.

**Table 4:** Texture parameters and syneresis of yogurts with and without curdlan and submitted or not to heat treatment. Values indicate mean ± standard-deviation.

Sample	Firmness (× 10^−3^ N)	Cohesiveness	Adhesiveness (N × mm)	Springiness (mm)	Gumminess (N)	Chewiness (N)	Syneresis (%)
A1	77 ± 2^c^	0.77 ± 0.02^a^	0.02 ± 0.01^c^	1.13 ± 0.01^a^^b^	5.9 ± 0.3^c^	6.7 ± 0.4^b^^c^^d^	49.3 ± 0.8^a^
A2	84 ± 8^c^	0.71 ± 0.07^a^^b^	0.04 ± 0.03^c^	1.00 ± 0.04^b^^c^	5.9 ± 0.1^c^	5.9 ± 0.3^c^^d^^e^	48.7 ± 0.4^a^
B1	83 ± 8^c^	0.66 ± 0.02^b^^c^	0.08 ± 0.03^c^	0.94 ± 0.04^c^	5.5 ± 0.7^c^	5.1 ± 0.7^e^	45.6 ± 0.9^b^
B2	129 ± 7^b^	0.47 ± 0.04^d^	0.4 ± 0.1^b^	0.97 ± 0.05^b^^c^	6.1 ± 0.2^b^^c^	5.9 ± 0.4^c^^d^^e^	38.2 ± 0.3^c^
C1	94 ± 9^c^	0.65 ± 0.02^b^^c^	0.16 ± 0.04^c^	1.2 ± 0.1^a^	6.0 ± 0.6^b^^c^	7.1 ± 0.3^b^^c^	26.4 ± 0.8^d^
C2	125.1 ± 0.6^b^	0.58 ± 0.02^c^	0.58 ± 0.02^a^^b^	1.01 ± 0.01^b^^c^	7.2 ± 0.2^b^	7.3 ± 0.2^b^	29.2 ± 0.7^d^
D1	128 ± 6^b^	0.46 ± 0.02^d^	0.68 ± 0.04^a^	0.96 ± 0.03^c^	5.8 ± 0.3^c^	5.6 ± 0.5^d^^e^	28.8 ± 0.2^d^
D2	160 ± 7^a^	0.59 ± 0.01^c^	0.5 ± 0.1^a^^b^	0.99 ± 0.06^b^^c^	9.5 ± 0.6^a^	9.3 ± 0.3^a^	35.8 ± 0.9^c^

^a–e^ Means within the same column with different letters are significantly different (p< 0.05).

Yogurts: A1) without curdlan or heat treatment; A2) without curdlan and with heat treatment; B1) with commercial curdlan and without heat treatment; B2) with commercial curdlan and heat treatment; C1) with pre-gelled commercial curdlan and without heat treatment; C2) with pre-gelled commercial curdlan and heat treatment; D1) with curdlan produced by *Agrobacterium* sp. IFO 13140 (pre-gelation method) without heat treatment; D2) with curdlan produced by *Agrobacterium* sp. IFO 13140 (pre-gelation method) with heat treatment.

It can be seen that the use of curdlan produced by *Agrobacterium* sp. IFO 13140 (pre-gelation method) produced significant alterations in the parameters of firmness, cohesiveness and adhesiveness of the yogurt, even without being subjected to heat treatment. This is due to the thickening potential of the material. Heat treatment caused major changes in these parameters, both for the yogurts with commercial curdlan and pre-gelled commercial curdlan, although the highest values for firmness, stickiness, gumminess and chewiness were obtained for the yogurt with curdlan produced by the microorganism. Heat treatment promotes the formation of a firm gel due to the intermolecular hydrophobic interactions that structure the system, making the yogurt more consistent, resulting in greater difficulty to separate in the mouth and making it denser during chewing, which therefore takes longer.

It was notable that the pre-gelled commercial curdlan formed a stronger gel in water, but had less effect on the texture parameters than that produced by *Agrobacterium* sp. IFO 13140 (pre-gelation method) when applied to the yogurt, which was not expected. Thus, it is likely that the latter has a greater ability to interact with water and the other components of milk, especially proteins, more efficiently stiffening the protein network formed after the fermentation process. As described before, the curdlan produced by the microorganism has the capacity to absorb 64% more water and 98% more oil than the pre-gelled commercial curdlan, with these parameters being important for products such as yogurts prepared with whole milk (3% fat).

The use of commercial curdlan without heat treatment did not produce many changes in the texture parameters or in the syneresis of the yogurts. This is because it was not effectively homogenized in the milk as it dispersed with greater difficulty, interfering little in the formation of the protein network during fermentation.

It was found that syneresis was reduced for the yogurt samples with curdlan, particularly the pre-gelled commercial variety and the curdlan produced by *Agrobacterium* sp. IFO 13140 (pre-gelation method). The latter displayed higher syneresis after the first 28 days of storage because its gel is less stable than the pre-gelled commercial curdlan. As such, the pre-gelled commercial curdlan demonstrated a greater ability to preserve the structure of the yogurt, avoiding rearrangements in the casein network due to the time of storage and avoiding whey expulsion. Martinez et al. [3] produced yogurts with added curdlan and achieved a significant reduction in the syneresis of the products compared to the control (without curdlan). The authors found that the high stability obtained by the yogurt with curdlan is attributed to interactions between the curdlan molecules, either between one another or with the proteins, which cause the formation of a more compact and continuous three-dimensional protein network, which effectively entraps the protein molecules and water in its structure.

**Fig 4:**
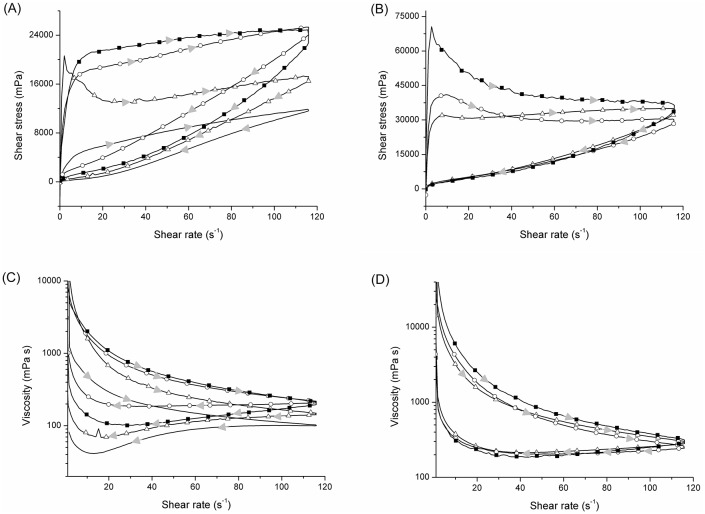
Flow curves of yogurt samples without heat treatment (A) with heat treatment (B); viscosity curves of yogurt samples without heat treatment (C) and with heat treatment (D). Yogurt without curdlan (–), with commercial curdlan (Δ), with pre-gelled commercial curdlan (○) and with curdlan produced by *Agrobacterium* sp. IFO 13140 (pre-gelation method) (■). The direction of the gray arrows indicates the ascendant and descendant curves.

Importantly, there was no difference between any of the texture parameters and the syneresis of yogurts without curdlan submitted or not to heat treatment. This was expected as the aim of treatment was to gel the curdlan and, therefore, the A2 sample was not submitted to rheological analysis. Fig 4 shows the flow curves (Fig 4A and 4B) and the viscosity curves (Fig 4C and 4D) for the yogurts produced with curdlan with and without heat treatment of the milk prior to fermentation.

All the yogurts behaved as non-Newtonian pseudo-plastic fluids, as their viscosity decreased due to the shear rate applied. It can be seen that the samples subjected to heat treatment of the milk had higher viscosity values across the entire shear rate analyzed due to the gelling of the curdlan. However, the decrease in viscosity versus the shear rate in the return data was much more significant for these samples. All the samples also exhibited thixotropic characteristics, due to the difference of tension and viscosity between the ascending and descending shear rate curves. The hysteresis observed is due to the breakdown of the gel structure formed by coagulation of the protein during fermentation, in the presence and absence of curdlan. Hysteresis is measured as the area between the ascending and descending curves, where the greater the area (when positive), the greater the thixotropic effect. Hysteresis, together with the other rheological parameters, are described in Table 5.

**Table 5:** Rheological parameters of yogurt samples with and without curdlan and submitted or not to heat treatment. Hysteresis, consistency index (K), flow behavior index (n) and yield stress (τ_0_). Values indicate mean ± standard-deviation.

*Sample*	*K (mPa s*^*n*^*)*	*n*	*τ*_*0*_ *(Pa)*	*Hysteresis (Pa s*^*-1*^*)*
A	98 ± 3^d^	0.997 ± 0.006^a^	2 ± 1^e^	362 ± 43^e^
B1	223 ± 26^d^	0.938 ± 0.001^b^	12 ± 1^d^	1088 ± 75^d^
B2	1685 ± 18^a^	0.662 ± 0.004^e^	30 ± 4^c^	2241 ± 39^b^
C1	922 ± 35^b^	0.73 ± 0.04^d^	12 ± 1^d^	1134 ± 92^d^
C2	—*	—*	36 ± 2^b^	2200 ± 49^b^
D1	467 ± 56^c^	0.87 ± 0.02^c^	10.9 ± 0.5^d^	1623 ± 36^c^
D2	—*	—*	75 ± 3^a^	3461 ± 10^a^

^a–e^ Means within the same column with different letters are significantly different (p< 0.05).

*It was not possible to calculate the parameters by the Oswald de Waele model.

In Fig 4B, the heat-treated samples of yogurt with pre-gelled commercial curdlan and curdlan produced by the microorganism displayed a peak in shear strain at the beginning of the growth of the deformation rate. This peak is related to the lack of homogeneity of the product, because during the gelling process of curdlan small lumps are formed, which are not necessarily noticeable to the palate, but which alter rheology. With the presence of these lumps, the tension needed for a small deformation is high, and as a result, it was not possible to calculate the K and n parameters for samples C2 and D2 by the Oswald de Waele model.

From the data of Table 5 it is clear that the higher viscosity samples in Fig 4C and 4D, which are those subjected to heat treatment, had more evident thixotropic characteristics (higher hysteresis), as they underwent a major reduction in apparent viscosity with time, in a rate constant with shearing. This is due to the breakdown of the organized yogurt structure when submitted to a determined strain. After remained stationary, the samples returned to their original state more quickly, but with lower viscosity during the return, being this recovery dependent on time. Therefore, the gel formed with heat treatment presented low stability, in particular the gel of the curdlan produced by the microorganism, corroborating with the syneresis data of the yogurt.

Curiously, it was observed that of the samples without heat treatment, that which had the greater viscosity in the increasing shear rate curve did not present higher K and n values, or in other words, the sample with pre-gelled commercial curdlan was greater than the curdlan produced by *Agrobacterium* sp. IFO 13140 (pre-gelation method) in the consistency index and flow behavior. This was due to the sharp drop in viscosity in the decreasing curve of the latter, revealing again the lower stability of the gel formed.

The results of the yield strength parameter (τ_0_) were consistent with the firmness of the yogurt, as the yogurt with curdlan produced by *Agrobacterium* sp. IFO 13140 with thermic treatment withstood the greatest tension before suffering deformation, followed by the yogurts with pre-gelled commercial curdlan and commercial curdlan with heat treatment. It is important to note that yield stress refers to the maximum stress that the material can bear before yielding (in the elastic deformation regime) and that the greatest τ_0_ values are those of the firmer samples. These samples displayed a minimum stress for deformation that was more difficult to break due to the increased organization and stiffening of the protein network formed, originating from the intermolecular hydrophobic interactions of the curdlan with heating.

## Conclusions

The characteristics of dispersion, gelation and the rheological properties of curdlan depended greatly on the recovery methods employed after its production, as the curdlan produced by *Agrobacterium* sp. IFO 13140 (pre-gelation method) and the commercial pre-gelled curdlan dispersed better in water, acted as thickeners and formed more homogeneous gels. The use of the pre-gelation method exerts a major influence on the size of the particles obtained and in the presence of NaCl, which contributes significantly to the dispersion and gelation characteristics described. However, the recovery method employed did not influence the structure of the polysaccharide. Even the pre-gelled commercial curdlans and those produced by the microorganism through the pre-gelation method had significantly different technological properties. The curdlan produced by *Agrobacterium* sp. IFO 13140 (pre-gelation method) showed a greater thickening and water and oil holding capacity than the pre-gelled commercial curdlan, while the latter demonstrated a considerably greater gelling capacity, that is related to degree of polymerization of the polysaccharide. As a consequence, although both types of curdlan increased firmness, viscosity and reduced the syneresis of yogurts, the curdlan produced by *Agrobacterium* sp. IFO 13140 (pre-gelation method) caused a greater increase in the parameters, but also showed a less stable gel formation than the pre-gelled commercial curdlan. The commercial curdlan did not influence the firmness and syneresis of yogurts tested. Nevertheless, the curdlans recovered by the pre-gelation method provided rigid gels with a stable structure, allowing improvements in the texture of a range of products, and therefore, has great potential for application in the food industry.

## Supporting information

S1 Appendix Curdlan interaction with aniline blue dye. (DOC)
