# Supplementary material for: Description of recovery method used for curdlan produced by Agrobacterium sp. IFO 13140 and its relation to the morphology and physicochemical and technological properties of the polysaccharide
Source: PLoS One. 2017 Feb 28;12(2):e0171469. doi: 10.1371/journal.pone.0171469 (PMC5330454; doi:10.1371/journal.pone.0171469)
Supplement: S1 Appendix — (DOC) [file pone.0171469.s001.doc]

**S1 Appendix**

**Curdlan interaction with aniline blue dye**

Solutions of the following concentrations of pre-gelled commercial curdlan and of curdlan produced by *Agrobacterium* sp. IFO 13140 (pre-gellation method) were prepared in NaOH 5 mmol L^-1^: 0, 50, 100 and 150 µg mL^-1^. These solutions were mixed with an equal volume of phosphate buffer (0.5 mol L^-1^, pH 7) containing 160 mg L^-1^ of aniline blue and kept for 120 min at room temperature. Then, the absorbance of the mixtures was measured at 590 nm. The analysis was performed in triplicate.


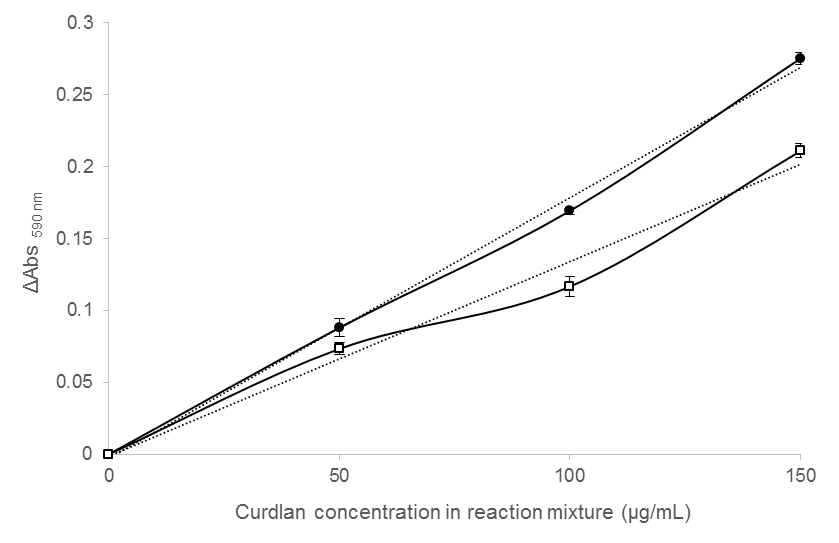


**Fig S1. Relationship between absorbance difference and concentration of curdlans.** Pre-gelled commercial curdlan (●) and curdlan produced by *Agrobacterium* sp. IFO 13140 recovered by the pre-gellation method (□).
